# Advances in Decellularization of Fish Wastes for Extracellular Matrix Extraction in Sustainable Tissue Engineering and Regenerative Medicine

**DOI:** 10.3390/bioengineering13020255

**Published:** 2026-02-23

**Authors:** Jady Lee Amarillas, Roger Dingcong, Lornie Grace Sabugaa, Maree Ivonne Kyla Domingo, Carl Angelo Samulde, Gerard Ian Pingoy, Abhel Ananoria, Roberto Malaluan, Ronald Bual, Gerard Dumancas, Arnold Lubguban

**Affiliations:** 1 Center for Sustainable Polymers, Iligan Institute of Technology, Mindanao State University, Iligan City 9200, Philippines; jadylee.amarillas@g.msuiit.edu.ph (J.L.A.); rogerjr.dingcong@g.msuiit.edu.ph (R.D.J.); roberto.malaluan@g.msuiit.edu.ph (R.M.); ronald.bual@g.msuiit.edu.ph (R.B.); 2 Department of Chemical Engineering and Technology, Iligan Institute of Technology, Mindanao State University, Iligan City 9200, Philippines; lorniegrace.sabugaa@g.msuiit.edu.ph (L.G.S.); mareeivonnekyla.domingo@g.msuiit.edu.ph (M.I.K.D.); carlangelo.samulde@g.msuiit.edu.ph (C.A.S.); gerardian.pingoy@g.msuiit.edu.ph (G.I.P.); abhel.ananoria@g.msuiit.edu.ph (A.A.); 3 Honors College, Henry E. and Shirly T. Frye Hall, Suite 110, North Carolina Agricultural & Technical State University, 1601 East Market Street, Greensboro, NC 27411, USA; gerard.dumancas@scranton.edu; 4 Department of Chemistry, New Science Building, North Carolina Agricultural & Technical State University, 1601 East Market Street, Greensboro, NC 27411, USA

**Keywords:** decellularization, fish tissue, tissue engineering, regenerative medicine, 3D printing

## Abstract

Decellularization removes immunogenic intracellular components of fish tissues while keeping the extracellular matrix (dECM) structure, mechanical integrity, and bioactivity. Fish-derived dECM retains native bioactive components, exhibiting high biocompatibility, low immunogenicity, and biodegradability, while supporting cell adhesion, proliferation, and tissue regeneration. Due to its abundance, minimal ethical concerns, and low zoonotic risks, fish wastes are emerging as sustainable sources of dECM, offering an eco-friendly alternative to mammalian biomaterials. This review highlights advances in decellularizing fish wastes such as skin, scales, bones, viscera, and swim bladders from species including tilapia, tuna, milkfish, carp, goldfish, and sturgeon. Physical, chemical, biological, and hybrid decellularization methods are assessed for cell removal, ECM preservation, and mechanical performance. Recent advances in polymer-dECM composites, crosslinking, and 3D bioprinting have significantly improved scaffold performance, making fish-derived dECM applicable for healing of wounds, regeneration of bone and cartilage, and repair of soft tissues. Despite its potential, challenges remain in optimizing perfusion rates, temperature variations, and tissue-specific protocols, as well as developing eco-friendly decellularization techniques using biodegradable reagents. Future perspectives include expanding decellularized fish tissue sources, innovating bio-inks for 3D bioprinting, and refining tissue-specific processing methods to maximize the potential of fish-derived dECM in regenerative medicine and tissue engineering.

## 1. Introduction

In recent years, global aquatic animal production has experienced a significant increase, reaching a new world high of 185 million tonnes (Mt) in 2022 from 110.7 million tonnes in the 1990s. Of this, 89% (164.6 Mt) was designated for direct human consumption, while the remaining 11% (20 Mt) was allocated for non-food applications. Among these non-food applications, about 83% (17 Mt) was processed into fishmeal and fish oil, whereas the remaining 4 Mt was used for diverse applications, including ornamental fish trade, fish bait, pharmaceuticals, pet food, and as feed in aquaculture or livestock farming [1]. This increase in global production raises concerns about the increase in waste generation, particularly for the food processing industry.

The processing of fish is essential to create a product of higher commercial value, stability, and quality; however, it comes at a cost of generating large amounts of waste. In this review, the term fish waste refers to underutilized by-products generated during fish processing, including skin, scales, bones, viscera, and other collagen-rich tissues that are typically discarded despite their high biomaterial potential. These wastes account for 30% to 70% of the total fish weight, varying species and processing techniques. Alarmingly, around 25% of this waste is improperly discarded into the environment [1,2,3].

Traditional waste management methods, such as disposal, fishmeal and oil production, fertilizers, and animal feed, generally yield low-value products and do not fully tap into the biochemical potential of fish-derived materials. Moreover, the simple disposal of these wastes can lead to various environmental problems, which can pose a threat to public health [1,4]. The typical methods of disposal of these wastes are landfill, incineration, and ocean dumping. Disposal by landfill causes anaerobic decomposition, releasing methane, ammonia, and hydrogen sulfide, which are harmful to the environment. Incineration, while a good option to eliminate methane emissions, is not economically viable as fish waste is high in moisture and low in energy. Lastly, ocean dumping causes oxygen depletion, increases in biochemical and chemical oxygen demand, aquatic organism suffocation, and the introduction of diseases into marine ecosystems [5,6]. Additionally, from an economic perspective, greater profitability lies in transforming fish waste into specialty commodities such as enzymes, peptides, and biopolymers, which have applications in the pharmaceutical and biomedical industries. This highlights the need for innovative and sustainable approaches to fish waste utilization, particularly for the development of high-value biomaterials [2].

**Figure 1:**
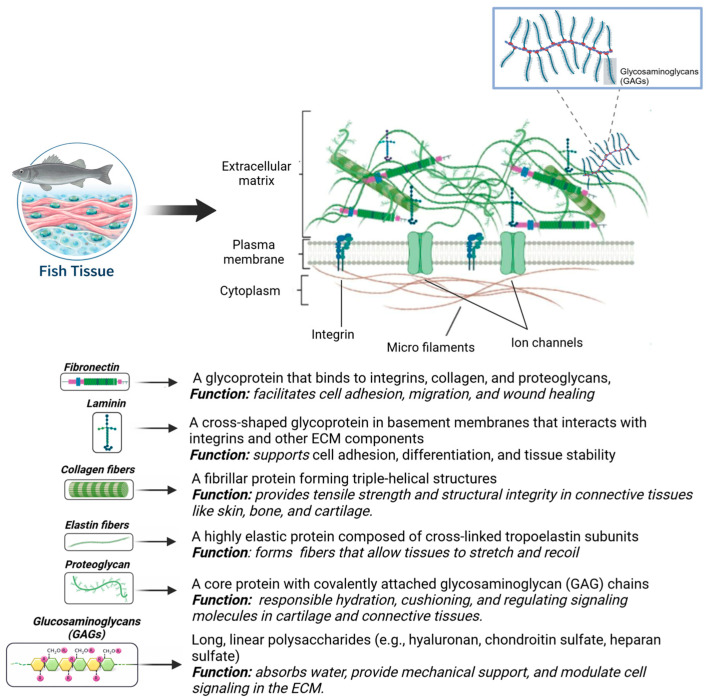
Protein compositions of fish ECM and their varied roles. Adapted from [9,10,11], under the Creative Commons CC BY 4.0 license.

Fish waste contains valuable biocompounds that can be used in biotechnological and pharmaceutical applications [1,3,7,8]. Viscera, for instance, contain proteins and enzymes which can be used to produce hydrolysates, while skin and bones are utilized to yield collagen and gelatin [4,8]. The extracellular matrix (ECM), primarily composed of fibrous proteins (e.g., collagen, elastin, fibronectin, laminin), glycoproteins, proteoglycans, and glycosaminoglycans (GAGs), plays a crucial role in maintaining structural integrity, cell signaling, and tissue function. Among these components, collagen is essential in forming the structural framework of the ECM (Figure 1), enhancing its significance as a key material for tissue engineering [9,10,11].

Collagen can be isolated and processed to create collagen scaffolds. These materials are utilized for the regeneration of nerves, bones, blood vessels, and skin due to their minimal immune response, excellent biocompatibility, and high biodegradability. Regardless, the limitations in mechanical strength and structural stability of pure collagen scaffolds have shifted research towards more advanced solutions, such as collagen-blended scaffolds and decellularized ECM (dECM) scaffolds [12]. There is an increasing interest in dECM due to the demand for organ replacement and the scarcity of donor organs. Furthermore, dECM offers high biocompatibility and biodegradability compared to synthetic ECM. This makes them well-suited for tissue engineering applications, including skin repair, bone and cartilage restoration, nerve grafting, cardiac tissue implants, and decellularized kidney scaffolds [13,14].

**Figure 2:**
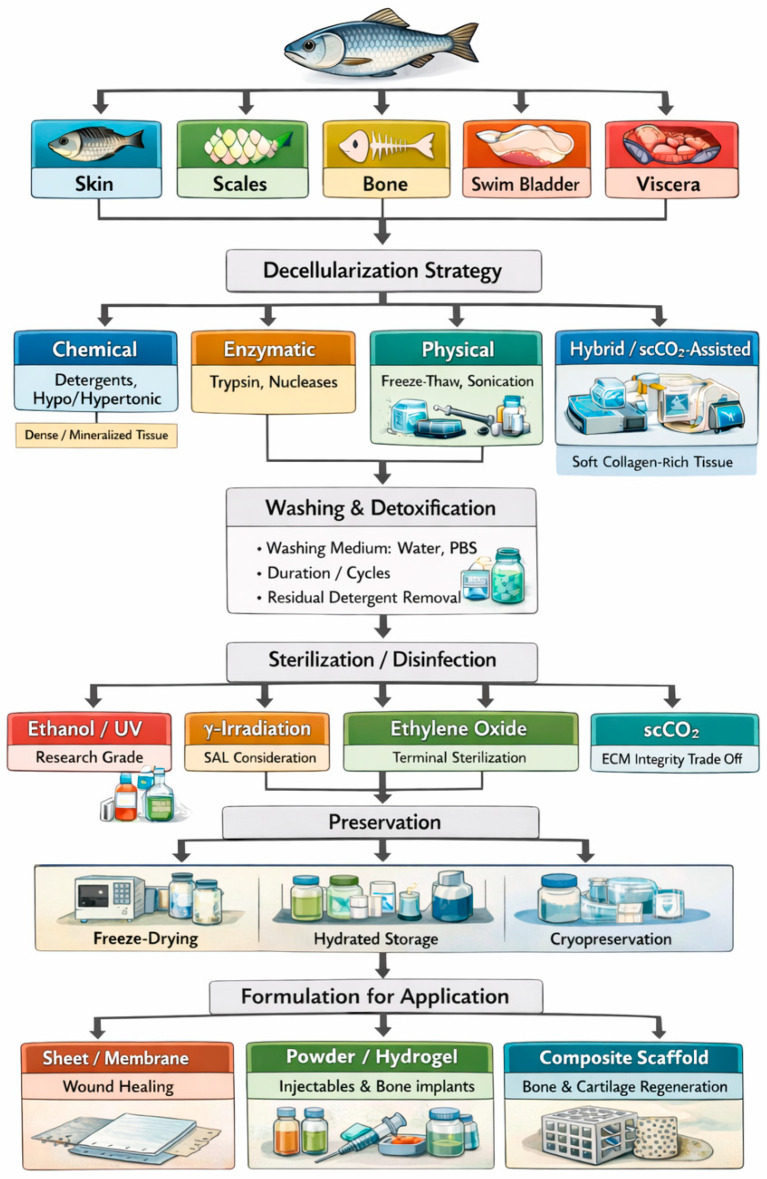
Schematic diagram for producing fish-waste-derived decellularized extracellular matrix (dECM), from tissue selection and processing to preservation and final biomedical applications.

Most decellularized tissue products come from human, bovine, and pig sources, with pig being the major source due to high availability and quantity. Contrary to pig, fish-derived dECM is still relatively new, as only around 7% of studies about decellularization in tissue engineering focused on fish sources from the period of 2022 to 2024 [13]. However, there has been an increase in fish tissue decellularization, primarily due to its advantages over mammalian counterparts. Fish-derived ECM poses a reduced risk of transmitting zoonotic pathogens, including bovine spongiform encephalopathy, swine influenza, and porcine endogenous retrovirus, while also facing fewer religious restrictions. Moreover, fish-derived biocompounds, such as collagen, have higher biodegradability, biocompatibility, and non-immunogenicity [13,15,16]. These aspects present a great opportunity for researchers to explore the potential of producing dECM from fish by-products, particularly fish waste. Figure 2 depicts the fish tissue decellularization process, where cells are eliminated through physical, chemical, biological, or hybrid treatments, leaving behind the dECM. The dECM then undergoes post-processing steps, including sterilization, functionalization, and preservation. Studies on fish-derived dECMs have highlighted their potential for biomedical applications, including wound healing, bone repair, and 3D bioprinting.

However, despite reports on using fish-derived dECM as scaffolds, there is no standardized protocol that exists yet in decellularizing fish tissues to date [17]. This review provides an overview and critical synthesis of fish tissue decellularization protocols, including sterilization, disinfection, preservation, and advancements in functionalization for fish-derived dECM. While the primary focus is on waste-derived fish tissues due to their relevance to sustainability and circular-economy frameworks, studies involving fish tissues more broadly are also considered when they provide relevant insights into decellularization strategies and ECM preservation. It also examines recent advancements in decellularization methods, their applications in biomedical and tissue engineering, and potential future directions in the field.

The literature reviewed in this manuscript was identified through searches of major electronic scientific databases, including PubMed, Web of Science, Scopus, and Google Scholar. Searches were conducted using combination of keywords such as fish-derived extracellular matrix, fish decellularization, decellularized extracellular matrix, tissue engineering and regenerative medicine. The review focused primarily on peer-reviewed articles published between 2015 and 2025, with earlier foundational studies included, where necessary, to provide context. Particular emphasis is given to the significant increase in publications observed between 2022 and 2024. Studies were selected based on relevance to fish-derived tissues and decellularization strategies for biomedical applications, while articles not directly related to ECM–based materials or scaffold development were excluded. Titles and abstracts were screened for relevance, followed by full-text assessment to identify studies suitable for inclusion in the qualitative synthesis presented in this review.

## 2. Fish Tissue Decellularization Techniques

Decellularization is a tissue engineering method that eliminates intracellular components of tissues while retaining the ECM, which supports structural integrity, biochemical signaling, and mechanical properties [18]. It plays a vital role in biomaterial development, providing a biocompatible scaffold for regenerative medicine, transplantation, and tissue repair, reducing immune rejection risks [19,20]. For effective decellularization, all immunogenic material must be removed while maintaining the ECM composition [21]. The methods used include chemical, enzymatic, and physical approaches, each offering specific benefits depending on the tissue type [22,23]. Often, a combination of methods is used to optimize cell removal and ECM preservation [24].

While standardized protocols for fish tissue decellularization are lacking [17], the general process follows four key steps to ensure scaffold preparation [23]. First, washing removes blood and debris, followed by the application of decellularization agents to dissolve cellular components [25]. Second, rinsing eliminates residual chemicals and cell fragments, reducing toxicity risks [18]. Third, sterilization ensures microbial safety. Finally, preservation methods such as freezing, lyophilization, or chemical treatments are applied to stabilize the ECM for future use [26]. Each step must be precisely controlled to safeguard the ECM’s delicate restructure and biological function.

Despite advancements in fish tissue decellularization, several challenges persist. Key concerns remain, such as maintaining the ECM structure, minimizing chemical residues, and enhancing mechanical strength [27]. Addressing these concerns requires a systematic evaluation of the available decellularization techniques and their trade-offs. Accordingly, the following sections examine the current physical, chemical, and biological approaches employed in fish tissue decellularization, highlighting their mechanisms, advantages, limitations, and suitability for different tissue types and biomedical applications.

### 2.1. Physical Treatment

Physical decellularization methods, such as freeze–thaw cycles, agitation, and sonication, rely on mechanical forces to remove cells while preserving the ECM. These methods reduce chemical use, maintain ECM structure, enhance agent penetration, and are cost-effective [28]. However, they may leave residual cells, risk ECM damage from excessive stress, and require careful optimization to prevent under- or over-treatment.

#### 2.1.1. Freeze–Thaw Process

Freeze–thaw cycles achieve decellularization by repeatedly subjecting tissues to freezing and thawing, leading to the formation of ice crystals within cells. As these crystals grow, they rupture cell membranes, causing cell lysis and detachment from the ECM [29]. This technique is straightforward, cost-efficient, and avoids harsh chemicals, minimizing the risk of ECM degradation [30]. However, excessive ice crystal growth can damage the ECM ultrastructure, which is why cryoprotectants like trehalose and dimethyl sulfoxide are often used to regulate crystal size [13,31]. Since freeze–thaw cycles alone may not fully remove cellular content, additional chemical treatments are typically required for complete decellularization. Moreover, excessive application of this method can weaken the ECM’s mechanical properties due to repeated structural stress from ice formation [32]. Which is why this physical method is best used as a preliminary treatment before tissues are exposed to chemical and/or enzymatic treatments [33,34,35,36]. Chen et al. demonstrated that zebrafish ventricles decellularized via freeze–thaw cycle retained essential components like collagen, elastin, and GAGs, supporting cardiac regeneration in mammalian models. However, residual DNA (1.6–1.9%) indicated incomplete decellularization, posing potential immune risks [37]. The findings suggest that while physical decellularization of fish-derived ECM holds promise for regenerative medicine, it may require additional treatments for full cell removal.

#### 2.1.2. Agitation

Agitation enhances decellularization by increasing tissue interaction with decellularizing solutions, improving detergent or enzyme diffusion, and accelerating cell removal. This is particularly useful for thick tissues with fewer blood vessels, where limited vascular pathways hinder the penetration of decellularizing agents into the tissue’s core [13,34,38,39,40]. Its advantages include faster processing, uniform cell removal, and improved efficiency. However, excessive agitation can damage fragile ECM, particularly in soft tissues like swim bladders, compromising structural integrity. Additionally, for thicker tissues, agitation alone may not achieve complete decellularization [41].

#### 2.1.3. Sonication

Sonication utilizes high-frequency sound waves to achieve cell removal through acoustic cavitation, a process in which ultrasonic waves generate microscopic bubbles that collapse, producing shear forces that break cell membranes and facilitate decellularization [7,17,42,43]. This technique effectively detaches cells with minimal contact time, preserving key ECM proteins and biomolecules [7,43]. However, excessive ultrasound exposure may degrade collagen, weaken ECM mechanics, and denature proteins, reducing bioactivity [44]. Without proper washing, nuclear materials can accumulate in the scaffold, affecting decellularization efficiency [7,43]. Despite these challenges, optimized sonication enhances efficiency while reducing chemical use, making it a valuable method for fish tissue decellularization.

Aron et al. and Baclayon et al. investigated sonication-assisted decellularization for ECM extraction from tilapia viscera and tilapia heads, respectively [7,17]. Both studies assessed the effectiveness of sonication in combination with detergents—sodium dodecyl sulfate (SDS) (0.3% and 1%) and TX-100 (0.1% and 0.3%)—for cell removal while preserving ECM integrity. Sonication at 40 kHz for 10 min significantly enhanced DNA removal, achieving 96.5% with 0.3% SDS (viscera) and 93.7% with 1% SDS (heads) by improving detergent penetration and reducing processing time. However, protein loss was slightly higher in sonicated samples, suggesting ultrasonic forces may partially disrupt ECM structure, as observed in SEM imaging. Despite this, FTIR and SDS-PAGE analyses confirmed collagen preservation, ensuring structural stability. High thermal resistance (>60 °C) and controlled enzymatic degradation further validated ECM suitability for biomedical applications. While residual SDS required extensive washing, both studies concluded that sonication effectively accelerates decellularization while maintaining ECM integrity, making it an efficient alternative to traditional agitation-based methods. While tilapia viscera and tilapia heads showed comparable decellularization efficiency, future research should focus on minimizing protein loss and optimizing detergent removal to optimize biocompatibility of sonication-assisted ECM scaffolds.

To date, only one study has reported the use of physical decellularization alone for fish tissues, in which zebrafish ventricles were subjected to repeated freeze–thaw cycles, resulting in partial decellularization with residual DNA levels approximately 1.6–1.9% [37], indicating incomplete cell removal and highlighting the limitations of physical methods when used without chemical or enzymatic support.

### 2.2. Chemical Treatment

Chemical decellularization in fish tissues commonly uses detergents, acids, bases, hypertonic or hypotonic solutions, or other solvents. While chemical treatments ensure thorough decellularization, excessive exposure may damage ECM integrity and leave toxic residues, requiring extensive rinsing. Optimized protocols often combine multiple agents to balance effectiveness and ECM preservation.

#### 2.2.1. Acidic and Basic Solutions

Acidic and basic solutions have been used in the decellularization of fish tissues. The mechanism behind acid-based decellularization primarily involves the hydrolysis of cellular structures, leading to cell lysis. Acids disrupt the cellular integrity by breaking down cell membrane components while promoting the solubilization of proteins. Widely used acids in fish decellularization include acetic acid [45] and nitric acid [46]. However, although acids can effectively reduce the presence of cellular debris, they may also catalyze the hydrolytic degradation of the ECM, which could compromise its mechanical strength and functional properties [47]. In most instances, combining acids with enzymes like pepsin is used to enhance the yield of extracted collagen and mitigate negative impacts on ECM integrity [20].

On the other hand, bases like sodium hydroxide are similarly effective in decellularization [45]. The use of bases helps to increase the pH of the solution, which can facilitate cell lysis through saponification and disruption of lipid components in the cell membrane. This method is particularly useful when combined with physical processes such as agitation or in combination with TX-100, which further enhances the removal of cellular remnants by increasing the contact between the base and the tissue [45].

Acidic and basic treatments effectively remove cellular debris, reducing immune rejection while preserving ECM components essential for regeneration [48]. Moreover, their concentrations can be adjusted to allow optimization for different tissue types [49]. However, prolonged acid and base treatment may reduce mechanical strength, while residual chemicals, if not thoroughly removed, can cause cytotoxic effects [50,51].

#### 2.2.2. Non-Ionic and Ionic Detergents

Detergents are amphiphilic molecules that aid in fish tissue decellularization by disrupting cell membranes. They are classified as non-ionic or ionic depending on their electrical charge. Non-ionic detergents, like TX-100, break lipid interactions while preserving ECM proteins, making them ideal for delicate tissues [33,52,53]. Their mild action retains biochemical signals for cell adhesion and growth, and they leave no toxic residues, enhancing biocompatibility [54]. However, they often require longer exposure and additional enzymatic or mechanical treatments to ensure complete cell removal [52,55,56].

In contrast, ionic detergents, like SDS [7,53,57,58], sodium deoxycholate and sodium orthovanadate [39,59], are more aggressive, solubilizing cellular and nuclear materials by disrupting membrane lipids and denaturing proteins except collagen [13]. This makes SDS highly effective for thick tissues like fish skin and scales; however, it also raises the risk of ECM damage by disrupting collagen structure and weakening mechanical properties.

Su et al. tested multiple protocols on Grass carp scales: 0.1% SDS treatment at 4 °C for 3 days, and a combination of TX-100, ethylenediaminetetraacetic acid (EDTA), and Nuclease at 4 °C for 3, 4, and 5 days. Results showed that while SDS treatment led to complete removal of cells, it had acquired serious damage to the ECM, poor mechanical properties, and a higher degradation rate compared to TX-100 treatments [55]. In general, the 4-day treatment proved to be the best protocol, ensuring optimal ECM composition and structure preservation, mechanical strength, cell biocompatibility, and osteogenic differentiation potential. Similarly, Arellano et al. compared TX-100 and SDS, testing various TX-100 concentrations at a 1:10 sample-to-solvent ratio. Their findings indicated that 0.1% TX-100 was most effective, outperforming SDS in structure and protein preservation [56].

#### 2.2.3. Hypotonic and Hypertonic Solutions

Hypotonic and hypertonic solutions aid in decellularization by using osmotic pressure to remove cells while preserving the ECM [60]. Hypotonic solutions, with lower solute concentrations than cells, cause water influx, leading to swelling and rupture. In contrast, hypertonic solutions draw water out, dehydrating cells and weakening adhesion, making debris removal easier [35,60,61].

Moreover, a combined approach enhances decellularization efficiency. Hypotonic treatment promotes cell lysis, followed by hypertonic treatment to remove residual debris and stabilize the ECM [62]. Sodium chloride is commonly used as a hypertonic solution, while Tris-buffer serves as a hypotonic agent in fish tissue decellularization [35,45,63]. By controlling osmotic conditions, they minimize the structural damage often caused by detergents [60]. However, optimizing concentration and exposure time is crucial, as improper balance can lead to incomplete decellularization or ECM degradation [62]. Additionally, enzymatic or detergent treatments are often needed for complete cell removal.

#### 2.2.4. Chelating Agents

Chelating agents like EDTA and ethylene glycol tetraacetic acid (EGTA) sequester divalent cations like calcium and magnesium by forming a stable complex around the metal ion, thereby reducing cell adhesion and promoting cell detachment. Although it has been proven that chelating agents alone are unsuccessful in complete decellularization, they make it easier for the detergents and other chemicals to disrupt and degrade cellular components [9]. This synergistic effect reduces the need for harsher treatments, preserving the ECM while improving the overall effectiveness of decellularization.

Table 1 presents various chemical decellularization protocols for fish tissues, comparing different methods, fish species, target tissues, procedures, and results. These protocols utilize a variety of chemical agents across different fish species, with detergents like TX-100 and SDS being the most commonly used due to their effectiveness. Other treatments, like chelating agents, hypotonic, and hypertonic solutions, primarily serve as supplementary components to enhance the process. In contrast, Table 2 introduces a more advanced approach that integrates both physical and chemical treatments to improve decellularization outcomes. For example, detergents are typically added with agitation to ensure thorough mixing. Sonication also assists in chemical decellularization by enhancing detergent penetration and accelerating the removal of cellular components. Meanwhile, a freeze–thaw cycle is frequently employed as a pre-treatment step before chemical application.

### 2.3. Biological Treatment

Biological decellularization of fish tissues uses enzymes to remove cells. In tissue decellularization, the common enzymes used are trypsin, nucleases, dispase, lipase, thermolysin, and α-galactosidase [9,13]. In fish tissues, recorded enzymes used include nucleases like DNase and RNase, which break down DNA and RNA for efficient removal after cell lysis [52,55,66]. Serine proteases, such as Trypsin, which break peptide bonds at lysine and arginine residues, aiding in protein digestion, including collagen in the ECM [33,59]. This treatment preserves growth factors and signaling molecules crucial for cell attachment and tissue regeneration [67]. However, it may struggle to fully remove cellular components and risks over-digestion, weakening the ECM by degrading collagen and GAGs [51,68].

A study noted that whole heart perfusion using trypsin alone led to only a 59% reduction in DNA content, whereas the combination with a chemical agent like TX-100 achieved a 90% reduction [47]. This stark difference highlights that relying solely on enzymatic processes could result in incomplete decellularization, which is crucial for subsequent applications in tissue engineering. Additionally, enzyme costs and storage requirements limit scalability. Combining enzymatic methods with physical or chemical techniques can improve efficiency while minimizing these challenges [69].

Table 3 and Table 4 show that no recorded studies have utilized biological treatment alone for fish tissue decellularization. In Table 3, enzymes are used in combination with chemical detergents, while Table 4 demonstrates the use of enzymes alongside chemical agents and physical methods, including freeze–thaw cycles and agitation.

### 2.4. Strategic Selection and Optimization Framework

The selection of an appropriate decellularization strategy for fish-derived tissues requires careful consideration of tissue-specific composition, structural complexity, and intended biomedical application. Variations in collagen content, mineralization, thickness, and cellular density across different fish tissues require tailored protocol designs rather than a one-size-fits-all approach. Moreover, decellularization efficiency must be balanced against preservation of ECM integrity and bioactivity, as overly aggressive treatments can compromise mechanical and biological performance.

To facilitate the selection of appropriate decellularization strategies for diverse fish-derived tissues, Table 5 provides a comprehensive strategic framework. This synthesis highlights the best-performing protocol of families identified in the recent literature.

Collectively, the physical, chemical, and biological decellularization strategies discussed above address cell removal with varying trade-offs in ECM preservation, residual toxicity, and mechanical integrity. These limitations highlight the need for tissue-specific protocol selection and optimization, as summarized in the preceding framework. However, even optimally fish-derived dECM remains vulnerable to microbial contamination and structural degradation, making appropriate sterilization and preservation essential to maintain bioactivity and translational viability. Accordingly, the next section focuses on sterilization and preservation strategies for fish-derived dECM.

## 3. Sterilization and Preservation of Fish-Derived dECM

Sterilization and preservation are critical post-decellularization processes that collectively determine the biological performance, reproducibility, and translational readiness of fish-derived dECM scaffolds in tissue engineering and regenerative medicine. Because these biomaterials are designed to directly interface with cells or host tissues, inadequate microbial control or improper storage can compromise scaffold bioactivity, provoke inflammatory responses, and invalidate experimental or clinical outcomes. At the same time, aggressive sterilization or preservation strategies can damage the collagen-rich ultrastructure, alter mechanical properties, and diminish bioactive cues that are essential for regenerative function.

In the context of fish-derived dECM, sterilization and preservation are inherently interdependent rather than sequentially independent steps. The choice of sterilization method, ranging from laboratory-scale disinfection to terminal sterilization techniques, must be evaluated in relation to the preservation state of the scaffold, as matrix water content, tissue thickness, and collagen organization strongly influence both microbial inactivation efficacy and ECM stability.

### 3.1. Sterilization Methods of Fish-Derived dECM

Across the fish-derived dECM literature, sterilization strategies range from laboratory-scale disinfection (e.g., ethanol or UV exposure) to clinically relevant terminal sterilization (e.g., gamma irradiation or ethylene oxide). Importantly, these approaches differ substantially in both sterility assurance and their impact on ECM structure, underscoring the need to interpret biological outcomes in light of the sterilization method employed.

#### 3.1.1. Gamma Irradiation

Among the sterilization strategies applied to fish-derived dECM, gamma (γ) irradiation emerges as the most consistently reported terminal sterilization method with clear translational relevance. Typical doses reported in fish-derived scaffolds range from 5 to 25 kGy, with irradiation applied to either freeze-dried or hydrated matrices prior to storage or implantation [16,45,71].

A key cross-study insight is that irradiation dose and hydration state are decisive variables. Comparative evaluations of 5, 10, and 25 kGy demonstrate that moderate doses (~10 kGy) effectively suppress microbial growth while preserving collagen fiber organization and staining intensity, whereas higher doses (≥25 kGy) can induce collagen fragmentation, reduced matrix density, and altered mechanical behavior [71]. These findings reconcile earlier reports in which γ-irradiation was considered both effective and potentially damaging; discrepancies largely arise from differences in dose selection, matrix water content during irradiation, and post-irradiation storage conditions.

From a tissue engineering perspective, this dose sensitivity is highly relevant. Collagen integrity governs scaffold stiffness, degradation kinetics, and cell–matrix interactions, all of which influence regenerative outcomes. Thus, γ-irradiation should be regarded not as a binary sterilization step but as a tunable processing parameter, ideally optimized alongside preservation method (e.g., freeze-dried versus hydrated) and validated using both sterility metrics and ECM functional assays.

#### 3.1.2. Ethylene Oxide and Chemical Disinfection

Ethylene oxide (EtO) sterilization is frequently applied to fish-derived dECM scaffolds, particularly for heat-sensitive tissues such as fish skin and scales [36,59]. EtO is typically applied to dry or semi-dry matrices, followed by aeration and storage under hydrated (e.g., PBS) or dry conditions. While EtO enables deep penetration and effective microbial inactivation at low temperatures, its application in fish-derived dECM studies is often reported without sufficient detail regarding exposure time, aeration duration, residual EtO quantification, or post-sterilization mechanical stability.

Conflicting outcomes reported for EtO-treated fish dECM can be reconciled by considering preservation format and application context. In hydrated storage, EtO-sterilized scaffolds have been successfully used for in vitro and in vivo regeneration [36]. Conversely, EtO treatment of freeze-dried fish skin matrices had been associated with structural destabilization under prolonged moist conditions, limiting suitability for wound coverage and long-term implantation [39]. These observations suggest that EtO compatibility is context-dependent, influenced by tissue composition, hydration state, and mechanical demands.

In contrast, ethanol-based disinfection (commonly 70–75% ethanol for 30–120 min, often combined with UV exposure) is widely used for fish dECM preparation prior to in vitro experiments [55,63,72]. While these methods are practical and preserve ECM bioactivity, they do not reliably inactivate spores and are not validated to clinical sterility standards. As such, ethanol and UV treatments should be interpreted as aseptic preparation techniques, suitable for laboratory studies but insufficient for implantable applications.

#### 3.1.3. Supercritical CO_2_-Assissted Processing

Recent studies exploring supercritical carbon dioxide (scCO_2_)–assisted decellularization highlight a promising direction for integrating decellularization, detergent extraction, and partial microbial reduction into a single process [72]. scCO_2_ processing is typically conducted at high pressures (≈200–350 bar) and moderate temperatures (35–45 °C), often with co-solvents such as ethanol to enhance penetration into dense fish tissues (e.g., swim bladder).

From a regenerative medicine standpoint, scCO_2_ processing offers two advantages: reduced residual detergent burden and preservation of mechanical integrity relative to aggressive chemical sterilants. However, despite its antimicrobial effects, scCO_2_ alone does not consistently achieve sterility assurance levels (SAL ≈ 10^−6^) required for implantable biomaterials. Consequently, its role may be best framed as a pre-sterilization or purification intensification step, rather than a standalone terminal sterilization strategy. Importantly, the effectiveness of sterilization is closely intertwined with the scaffold’s preservation state. Factors such as hydration, freezing, or drying not only influence microbial inactivation but also modulate collagen stability, mechanical integrity, and bioactivity. Consequently, selecting an appropriate preservation strategy is critical to ensure that fish-derived dECM maintains structural and functional fidelity following sterilization, as discussed in the next section.

### 3.2. Preservation Methods of Fish-Derived dECM

Within the existing body of research regarding fish-derived dECM, preservation methods recorded include freeze-drying, hydrated storage, and cryogenic preservation. These methods not only affect shelf-life and handling, but also the scaffold’s response to subsequent sterilization and its ability to retain structural integrity and bioactivity upon rehydration or implantation.

#### 3.2.1. Freeze-Drying/Lyophilization

Freeze-drying is the most widely employed preservation technique for fish-derived dECM across tissue types, including skin, scales, cartilage, and swim bladder [16,45,55,58,71]. Typical workflows involve freezing at −20 to −80 °C, followed by primary and secondary drying under vacuum, producing dry, porous scaffolds suitable for room-temperature storage and terminal sterilization.

Synthesizing across studies, freeze-drying can preserve collagen architecture and pore structure when freezing rate, residual moisture content, and rehydration protocol are carefully controlled. Histological and biochemical analyses demonstrate retained collagen organization after rehydration (commonly in saline or PBS for 10–30 min), supporting effective cell infiltration and matrix remodeling [71]. However, freeze-drying can also reduce mechanical strength and accelerate degradation if not paired with reinforcement or crosslinking strategies.

Critically, preservation outcomes are highly sensitive to process parameters, including freezing temperature, drying duration, residual moisture targets, and rehydration conditions, yet these variables are rarely reported in sufficient detail. This lack of standardization limits reproducibility and complicates cross-study comparison of biological performance.

#### 3.2.2. Hydration and Cryogenic Preservation

Hydrated storage (e.g., immersion in PBS or saline at 4 °C) is commonly used for EtO-sterilized fish-derived dECM intended for immediate or short-term use [36]. This approach preserves flexibility and avoids rehydration artifacts, making it advantageous for soft-tissue applications such as wound healing. However, long-term wet storage increases risks of enzymatic degradation and microbial contamination unless packaging, sterility monitoring, and storage duration are rigorously controlled.

Cryogenic preservation using cryoprotectant solutions (e.g., dextran-, sucrose-, or raffinose-based formulations) has also been applied to fish-derived dECM to minimize ice-crystal damage and maintain ECM ultrastructure [70]. While effective at preserving structure, reliance on cold-chain logistics and limited shelf-life poses challenges for large-scale clinical translation.

### 3.3. Clinical Translation Considerations for Fish-Derived dECM

Biocompatibility remains a key concern when transitioning from in vitro studies to in vivo applications, particularly given the phylogenetic differences between fish and humans. Nevertheless, accumulating evidence suggests that fish-derived dECM exhibits favorable host compatibility. For example, Heo et al. demonstrated that fish-derived dECM supported wound healing by promoting the expression of regenerative skin phenotype markers, including type I and III collagen and α-smooth muscle actin (α-SMA) [73]. This observation is further supported by Lee et al. [74], who reported that tilapia skin–derived dECM, enriched with native extracellular matrix constituents such as collagen and proteoglycans, enhances cell adhesion and mechanotransduction.

Despite these encouraging biological attributes, translation toward clinically relevant applications remains constrained by several unresolved challenges related to sterilization, preservation, and post-processing validation. Across the literature, these steps are often treated as secondary to decellularization, even though they exert a comparable influence on scaffold safety, reproducibility, and biological performance.

One of the most persistent gaps in the literature is the lack of standardized sterility validation. While many studies specify a sterilization or disinfection method, few report quantitative measures such as bioburden reduction, sterility assurance levels (SAL), or endotoxin content—metrics that are routinely expected for implantable biomaterials. This omission complicates cross-study comparison and obscures the true translational readiness of fish-derived dECM scaffolds, particularly for applications involving prolonged implantation or compromised wound environments.

Residual processing agents represent a second critical concern. Ionic detergents such as SDS are commonly employed during fish-derived dECM decellularization, yet residual levels are inconsistently quantified. Sterilization and preservation steps can further concentrate or redistribute these residues, directly influencing cytocompatibility, inflammatory response, and degradation behavior. Without systematic assessment of residual detergents and sterilants, it remains difficult to disentangle scaffold-driven regenerative effects from artifacts introduced during processing.

Batch variability also poses a significant challenge for clinical translation. Fish-derived raw materials vary according to species, tissue type, season, and upstream processing conditions, all of which can affect collagen composition, lipid content, and initial microbial load. However, these variables are rarely incorporated into sterilization or preservation decision-making, limiting reproducibility and complicating scale-up efforts. Greater transparency regarding tissue sourcing and preprocessing conditions would therefore strengthen the reliability of reported outcomes.

Finally, the relationship between preservation strategy and functional performance remains underexplored. Preservation methods such as freeze-drying, hydrated storage, or cryogenic preservation alter pore architecture, mechanical properties, and degradation kinetics, yet these changes are seldom correlated with in vivo regenerative outcomes. As a result, preservation is often discussed in terms of convenience or shelf-life rather than as a design variable that shapes tissue integration and remodeling.

Addressing these challenges will require a shift toward more systematic and transparent reporting practices that integrate sterilization parameters, preservation conditions, and post-processing validation with biological performance metrics. Rather than prescribing a single optimal protocol, future progress in fish-derived dECM research will depend on harmonized reporting frameworks that enable meaningful comparison across studies while allowing flexibility for tissue- and application-specific optimization. Such an approach is essential to fully realize the potential of fish-derived dECM as a sustainable and clinically relevant platform for tissue engineering and regenerative medicine.

## 4. Biomedical Applications of Fish-Derived dECM

Fish-derived dECM has been most extensively explored for soft tissue repair, particularly wound healing and skin regeneration; however, reported biological outcomes vary markedly across studies [13,73,75]. As summarized in Table 6, these variations are closely linked to fish species, tissue source, decellularization chemistry, and post-processing conditions, indicating that biological performance is highly context-dependent rather than uniform.

Across both in vitro and in vivo models, fish skin–derived dECM has been reported to support fibroblast migration, keratinocyte proliferation, and matrix remodeling—processes central to cutaneous repair. These responses are commonly associated with the preservation of collagen-rich fibrillar networks and GAGs [33], which facilitate cell–matrix interactions and provisional matrix formation. Several studies further report the retention of residual bioactive components, including omega-3 polyunsaturated fatty acids such as docosahexaenoic acid (DHA) and eicosapentaenoic acid (EPA) [96,97,98], which may contribute to inflammatory modulation and angiogenic signaling. Importantly, as reflected in Table 6, the influence of decellularization on these biological responses can be broadly attributed to three interacting parameter classes: (i) chemical aggressiveness (e.g., detergent type and concentration), (ii) exposure intensity (treatment duration and repetition), and (iii) tissue-specific susceptibility (fish species, tissue thickness, and native matrix density). The extent to which these biochemical features are retained is therefore strongly influenced by the combined effects of detergent selection, exposure duration, and tissue morphology.

Quantitative outcomes reported for fish-derived dECM in wound models frequently include accelerated wound closure rates, increased re-epithelialized surface area, and thicker or more mature granulation tissue formation when mild to intermediate decellularization protocols are employed. In contrast, studies using aggressive chemical or enzymatic treatments consistently report partial loss of GAGs and disruption of collagen organization, which can attenuate angiogenic responses and delay matrix remodeling [90,95]. Mechanistically, increased chemical aggressiveness accelerates cellular component removal but concurrently disrupts sulfated GAGs and collagen structure, whereas milder protocols preserve matrix-associated bioactivity at the expense of incomplete cellular clearance. Table 6 highlights that these outcomes reflect protocol-dependent trade-offs rather than uniform biological trends.

Beyond skin regeneration, fish-derived dECM has also been investigated for bone and cartilage repair, although the available evidence base is comparatively limited. As summarized in Table 6, fish-derived bone and scale matrices have been shown to support osteogenic differentiation and mineralization in vitro, while fish cartilage–derived dECM supports chondrocyte attachment and differentiation in select studies [75,84]. However, decellularization-induced loss of mineral organization or sulfated GAGs has been reported to compromise mechanical integrity and compressive performance, underscoring the heightened sensitivity of load-bearing applications to treatment parameters [99].

### 4.1. Wound Healing and Skin Regeneration

Tissue repair involves a coordinated sequence of inflammation, tissue regeneration, and remodeling. Fish-derived dECM materials have been shown to promote cellular migration, proliferation, and matrix remodeling, making them attractive scaffold candidates for wound healing and skin regeneration applications. These materials typically contain high levels of GAG, which promote granulation tissue formation and vascularization, thereby facilitating wound closure [37,100,101,102]. In addition, fish skin ECM is rich in omega-3 polyunsaturated fatty acids, particularly DHA and EPA [103]. These bioactive components are known to modulate inflammatory responses, regulate pro-inflammatory cytokine activity [41], enhance angiogenesis [33], and support granulation tissue formation [42,43]. Nevertheless, excessive EPA exposure has been reported to impair certain aspects of wound healing, such as collagen organization, indicating that an appropriate balance of omega-3 polyunsaturated fatty acids remains essential for optimal tissue repair outcomes [44].

Current studies consistently demonstrate the effectiveness of fish-derived dECM in accelerating wound healing and promoting skin regeneration [103,104]. Several reports indicate reduced healing time and significantly higher wound closure rates compared with control treatments [46,48]. For example, Lin et al. developed a bioprinted textile incorporating fish skin dECM with a tunable porous architecture, which enabled adequate air permeability and controlled delivery of bioactive molecules to enhance wound healing [105].

Beyond bioprinted constructs, acellular fish skin grafts have also shown strong regenerative performance. These grafts promote granulation tissue formation and collagen deposition, leading to enhanced re-epithelialization [40], and exhibit faster epithelial regeneration with reduced wound contraction compared with other skin graft materials [106]. Importantly, acellular fish skin grafts present a low risk of viral disease transmission, even when processed using minimal decellularization protocols, allowing the preservation of native ECM architecture and bioactive components [104]. This combination of biosafety, structural integrity, and biological activity contributes to the growing clinical interest in fish-derived dECM for wound care applications.

Across studies, wound healing and skin regeneration consistently emerge as the most successful biomedical applications of fish-derived dECM [70]. This favorable performance reflects a strong alignment between the intrinsic properties of fish skin ECM—rich in collagen, GAGs, and bioactive lipids—and the biological requirements of soft tissue repair. Unlike load-bearing tissues, skin regeneration imposes relatively low mechanical demands, enabling fish-derived dECM to function effectively even with limited post-processing and simpler decellularization protocols [107]. Nevertheless, further optimization remains necessary to address variability arising from fish species, tissue thickness, and processing conditions, to fully maximize the therapeutic potential and reproducibility of fish-derived dECM in wound healing and skin regeneration.

### 4.2. Bone Regeneration

Bone regeneration has attracted significant research interest due to the limited intrinsic healing capacity of osseous tissue, which arises from the need for rapid vascularization, mineralized matrix formation, and sustained mechanical stability under load [108]. In tissue engineering, DBM scaffolds are widely used to facilitate and enhance bone formation by providing osteoinductive and osteoconductive cues [109]. Compared with conventional mammalian-derived DBM, fish-derived DBM has emerged as a promising alternative owing to its favorable bioactivity and compositional characteristics, which can support scaffold-mediated regeneration while offering advantages in sustainability and biosafety [110].

Fish-derived DBM has been shown to support cell adhesion and proliferation [52] and to stimulate osteogenic differentiation, as evidenced by increased alkaline phosphatase activity, mineral deposition, and expression of osteogenic markers [110,111,112,113]. In addition, the presence of naturally occurring hydroxyapatite in fish scales and certain collagen-rich membranes contributes to improved mechanical reinforcement and promotes stem cell–mediated bone formation [52,91,92]. These findings indicate that fish-derived mineralized matrices can provide biologically relevant cues for bone regeneration when key structural features are preserved.

Despite these advantages, the application of fish-derived dECM in bone regeneration reveals important performance limitations. While osteoinductive responses are commonly observed in vitro and in vivo, maintaining sufficient mechanical stability during early-stage bone healing remains challenging. This limitation is particularly evident when decellularization or demineralization processes disrupt the native mineral–collagen organization, resulting in reduced load-bearing capacity and accelerated scaffold degradation. These observations highlight that biological activity alone is insufficient to ensure functional bone regeneration and underscore the need for additional structural reinforcement strategies, particularly for load-bearing applications.

### 4.3. Cartilage Repair

Cartilage repair presents an even greater challenge than bone regeneration due to the avascular nature of cartilage, its low cellularity, and the limited regenerative capacity of chondrocytes. In addition, inappropriate differentiation toward hypertrophic phenotypes can compromise long-term repair outcomes [40]. Fish cartilage–derived dECM has therefore attracted attention as a biomimetic scaffold capable of supporting chondrogenic responses while preserving tissue-specific extracellular matrix cues.

Li et al. demonstrated that a decellularized sturgeon cartilage ECM scaffold effectively repaired cartilage defects while significantly reducing chondrocyte hypertrophy, suggesting favorable regulation of chondrogenic differentiation pathways [40]. Similarly, Khajavi et al. reported that fish cartilage–derived dECM scaffolds promote cell attachment, migration, and differentiation toward chondrocyte-like phenotypes [58]. These studies indicate that fish-derived cartilage matrices can support key biological processes required for cartilage repair when appropriate decellularization protocols are employed.

However, decellularization of cartilage-derived matrices is particularly sensitive to the loss of sulfated GAGs, which are critical for compressive strength, hydration, and shock-absorbing function. Although collagen content in fish cartilage–derived dECM scaffolds often remains comparable to that of native cartilage, a pronounced reduction in GAG content following decellularization has been consistently reported [114]. This depletion can compromise the long-term mechanical performance of cartilage scaffolds under physiological loading conditions, even when initial cellular responses are favorable.

To fully realize the clinical potential of fish-derived dECM for cartilage repair, strategies that preserve key ECM components, regulate degradation behavior, and restore compressive mechanical integrity are essential. Approaches such as polymer reinforcement and composite scaffold fabrication represent promising solutions to address these limitations and are discussed in the following section.

### 4.4. Comparative Performance and Application-Driven Insights

The biomedical performance of fish-derived dECM is strongly application-dependent and governed by the alignment between preserved matrix bioactivity and the mechanical demands of the target tissue (Table 6). Rather than exhibiting uniform behavior across applications, fish-derived dECM demonstrates distinct performance windows shaped by tissue type, decellularization strategy, and application-specific functional requirements.

Across reported studies, soft tissue applications—particularly wound healing and skin regeneration—consistently show favorable biological outcomes. Fish skin–derived dECM supports key processes such as cell adhesion, proliferation, re-epithelialization, and granulation tissue formation when native extracellular matrix components, including collagen and GAGs, are preserved through mild to intermediate decellularization protocols [73]. These applications impose relatively low mechanical and structural demands, allowing fish-derived matrices to perform effectively despite limited tensile strength and comparatively faster degradation. As a result, soft tissue repair currently represents the most translationally mature application domain for fish-derived dECM.

In contrast, bone and cartilage regeneration reveal a divergence between biological compatibility and functional performance. While fish-derived dECM and demineralized matrices have been shown to support osteogenic and chondrogenic cellular responses, their use in mechanically demanding environments is constrained by insufficient structural stability and sensitivity to decellularization-induced matrix loss [17]. In cartilage repair, reductions in sulfated glycosaminoglycan content following decellularization compromise compressive strength and shock-absorbing capacity [115], which are critical for long-term functional outcomes. These observations imply that favorable cellular responses alone are insufficient to ensure success in load-bearing or mechanically complex applications.

Collectively, these findings indicate that the translational success of fish-derived dECM is governed less by intrinsic bioactivity alone than by the extent to which material properties are aligned with tissue-specific mechanical and functional demands. While soft tissue applications can often tolerate limited mechanical reinforcement, load-bearing and mechanically complex tissues require additional strategies to stabilize matrix architecture, regulate degradation, and preserve critical extracellular matrix components. These constraints motivate, but do not presuppose, the post-decellularization engineering approaches discussed in the following section, which aim to address identified performance limitations rather than redefine the application space of fish-derived dECM.

## 5. Advances in Post-Processing of Fish-Derived dECM

In response to the application-dependent limitations identified in Section 4, a range of post-processing strategies has been explored to enhance the functional performance of fish-derived dECM. The biological performance of dECM scaffolds is governed by the preservation of native matrix architecture, adequate mechanical integrity, and controlled degradation behavior [116]. While fish-derived dECM exhibits inherently favorable bioactivity, decellularization and subsequent processing steps can compromise these attributes, particularly in mechanically demanding or long-term biomedical applications. Consequently, post-processing interventions play a critical role in restoring and tailoring the structural, mechanical, and biological properties of fish-derived dECM to meet application-specific requirements.

Post-decellularization modifications, including surface crosslinking, chemical stabilization, and polymer hybridization, are also widely employed to improve structural robustness, regulate degradation kinetics, and enhance handling and processability. Table 7 provides a comparative summary of these post-processing strategies, highlighting the specific limitations they address, the reported quantitative improvements in mechanical and functional performance, and the associated trade-offs. Collectively, these approaches enable the rational tuning of scaffold mechanics, bioactivity retention, and translational feasibility without negating the intrinsic biological cues of the native matrix, thereby bridging the gap between the biological advantages of fish-derived dECM and the performance demands of regenerative medicine and tissue engineering applications.

### 5.1. Crosslinking for Surface Modification

Surface modification strategies, particularly crosslinking, play a critical role in enhancing the performance of dECM scaffolds. Decellularization processes can disrupt native ECM architecture, accelerate degradation, and reduce mechanical strength, thereby limiting long-term functionality in bio-medical applications. Crosslinking addresses these challenges by introducing covalent or ionic bonds between polymer chains, which reinforce structural integrity, stabilize mechanical properties, and regulate degradation behavior [116]. In addition, residual immunogenic components may persist after decellularization and potentially trigger adverse immune responses [119]; crosslinking of collagen fibers has been shown to mitigate such risks while improving thermal stability, mechanical resilience, and overall bio-compatibility [120,124].

Both physical and chemical crosslinking techniques have been investigated to extend ECM durability while preserving cytocompatibility. Physical approaches, such as dehydrothermal (DHT) treatment, induce intermolecular covalent bonding through controlled heat and vacuum exposure, thereby enhancing matrix stability without introducing chemical residues. Chemical crosslinkers are more commonly employed due to their tunability and effectiveness. Among these, 1-ethyl-3-(3-dimethylaminopropyl)-carbodiimide (EDC) in combination with N-hydroxysuccinimide (NHS) has emerged as a safer alternative to traditional agents such as glutaraldehyde (GA) [120,121]. Although GA effectively improves ECM strength, its cytotoxicity and potential for long-term adverse effects limit its suitability for biomedical use [137].

EDC functions as a zero-length crosslinker, facilitating the formation of stable amide bonds between carboxyl and amine groups without leaving harmful residues [37]. NHS further enhances crosslinking efficiency by generating reactive intermediates that are water-soluble and readily removed through repeated rinsing steps [138]. Lan et al. demonstrated the effectiveness of EDC/NHS crosslinking in fish swim bladder–derived ECM, where amide bond formation improved mechanical strength, thermal stability, and resistance to enzymatic degradation without residual crosslinker contamination [122]. Scanning electron microscopy revealed a well-preserved porous structure conducive to cell interaction, while in vitro biocompatibility assays confirmed the absence of inflammatory responses and enhanced cell adhesion and proliferation. However, excessive crosslinking was shown to reduce scaffold porosity, which may impede cell infiltration and tissue integration [122]. These findings highlight the importance of optimizing crosslinking density to balance mechanical reinforcement with biological accessibility.

Natural crosslinking agents have also attracted attention as potentially safer and more biologically favorable alternatives. Genipin (GP) is the most widely studied natural crosslinker, although its high cost and variable efficacy can limit large-scale application [23,120,122]. Procyanidins (PC), a class of naturally occurring polyphenols, have emerged as a promising alternative due to their antioxidant, anti-calcification, anti-inflammatory, and anti-tumor properties [120]. To date, the application of procyanidins as crosslinking agents for fish-derived dECM has not yet been reported, highlighting an unexplored opportunity for improving mechanical stability and biocompatibility using bio-derived, low-toxicity crosslinkers. Exploring such natural crosslinking strategies may further enhance the translational potential of fish-derived dECM in regenerative medicine.

### 5.2. Polymer–dECM Composites Fabrication

Polymer–dECM composite fabrication has emerged as an effective strategy to overcome the inherent mechanical weakness, rapid degradation, and limited processability of fish-derived dECM. Among composite systems, hydrogels are particularly attractive due to their high-water content, interconnected porosity, and capacity to mimic the native extracellular environment. These properties support cell proliferation, migration, and nutrient diffusion, making hydrogel systems widely explored in soft tissue regeneration contexts [105].

Hydrogels can be formulated from a broad range of natural and synthetic polymers, including alginates, chitin, collagen, polylactic acid, and polyglycolic acid. While many of these polymer systems and design principles are broadly applicable across mammalian and non-mammalian dECM platforms, their integration with fish-derived dECM introduces tissue-specific biochemical cues that can enhance biological performance while reducing reliance on aggressive chemical modification. Accordingly, the discussion below distinguishes general composite design concepts from examples explicitly demonstrated using marine-derived matrices.

Several studies have demonstrated the advantages of polymer–dECM hybrid systems incorporating fish-derived materials. Lin et al. combined fish skin dECM with methacrylic anhydride (MA) and alginate (Alg) to improve mechanical stability and processability [126,127,128,129]. Using a 3D bioprinting platform equipped with long-wavelength UV lamps, the authors fabricated hydrogel microfibers assembled into three-dimensional textile-like structures [105]. These constructs exhibited porous and rough surface morphologies with interconnected internal pores, enabling effective loading and controlled release of bioactive agents for wound healing applications.

In another example, Jia and Wang developed a multi-component composite hydrogel by combining small intestinal submucosa (SIS) decellularized matrix, fish collagen (FC), and methacrylated gelatin (GelMA) [131]. Although SIS-based systems are not marine-derived, this study illustrates generalizable composite design principles, such as complementary polymer interactions and tunable gelation behavior, that are directly applicable to fish-derived dECM systems when appropriate marine-based matrices are substituted.

Beyond bulk hydrogel systems, polymer–dECM composites also enable controlled incorporation of cells and bioactive compounds. Ren et al. fabricated hydrogels composed of fish liver dECM and GelMA, loaded with induced pluripotent stem cell–derived hepatocytes (iPSC-heps), demonstrating improved hepatic regeneration in vivo [132]. Similarly, Lin et al. incorporated antibacterial curcumin and basic fibroblast growth factor into fish dECM-based hydrogels, achieving enhanced wound repair outcomes through synergistic biological signaling [105]. These examples show polymer–dECM composites can simultaneously provide mechanical reinforcement and functional bioactivity.

In addition to hydrogel-based systems, nanocomposite approaches have been explored to further enhance the mechanical and biological performance of fish-derived materials. Lima-Verde et al. reported collagen nanofibers derived from tilapia skin, scales, and spine, which were combined with various biomaterials to form nanocomposites exhibiting improved mechanical strength and biocompatibility [139]. Recent progress in this area has expanded toward the incorporation of bioactive inorganic nanophases, surface-functionalized nanoparticles, and hierarchical fiber architectures to modulate stiffness, degradation kinetics, and cell–matrix interactions [140]. These nanostructured systems highlight the potential of nanoscale reinforcement strategies that are explicitly compatible with marine-derived dECM sources.

### 5.3. Three-Dimensional Printing

Three-dimensional (3D) printing and related biofabrication techniques should be viewed not as biomedical applications per se, but as enabling fabrication strategies that support the practical deployment of fish-derived dECM across diverse tissue targets. Conventional scaffold fabrication methods typically rely on porous biocompatible polymeric materials, which often require surface modification to enhance cell adhesion and proliferation [141]. In contrast, 3D printing enables the controlled assembly of scaffolds with defined architectures by spatially organizing cells and bioactive components within hydrogel matrices [13,105,135]. This approach allows precise control over microstructural features such as pore size, porosity, and spatial organization, thereby supporting application-specific scaffold design.

The use of dECM-based bio-inks has attracted increasing attention due to their inherent bioactivity and tissue-specific biochemical cues. However, fish-derived dECM alone typically exhibits low viscosity and limited mechanical integrity, posing challenges for maintaining print fidelity and structural stability during extrusion-based printing. To overcome these limitations, fish-derived dECM is frequently combined with natural or synthetic polymers to improve rheological properties and mechanical performance [32]. For example, Lin et al. developed a hybrid bio-ink composed of fish skin dECM and MA, incorporating curcumin and fibroblast growth factor to enhance biological functionality while maintaining printability and scaffold integrity [105]. Similarly, Bui et al. formulated a composite bio-ink combining fish-derived dECM with methacrylated hyaluronic acid, crosslinked using divinyl sulfone, achieving improved printability and tunable hydrogel properties [125]. In this system, swelling behavior and mass erosion rates varied proportionally with fish-dECM content, illustrating the importance of compositional balance in optimizing scaffold performance.

More advanced biofabrication strategies further expand the functional utility of fish-derived dECM by integrating composite formulation with in situ stabilization techniques. Ren et al. employed microfluidic 3D bioprinting coupled with in situ photopolymerization to fabricate composite scaffolds composed of fish-derived dECM, GelMA, and induced pluripotent stem cell–derived hepatocytes (iPSC-heps) [132]. The use of in situ photopolymerization preserved scaffold architecture and bioactivity, resulting in enhanced biocompatibility, improved cell adhesion, and increased survival in a mouse model of acute liver failure [136]. These findings demonstrate that biofabrication approaches primarily address the processing-related limitations of fish-derived dECM rather than defining its biomedical application space.

### 5.4. Engineering Trade-Offs and Design Considerations

The comparative analysis summarized in Table 7 indicates that post-processing strategies are essential for enhancing the functional performance of fish-derived dECM, but these approaches inherently introduce engineering trade-offs that must be carefully managed. Crosslinking techniques can substantially improve mechanical stability and regulate degradation behavior; however, excessive crosslinking frequently reduces scaffold porosity and limits cell infiltration, thereby compromising biological integration [17,142]. Similarly, polymer–dECM composite systems offer a means to reinforce mechanical properties while retaining tissue-specific biochemical cues, yet increased polymer content and processing complexity may dilute matrix-derived bioactivity or alter degradation kinetics [23,99].

More advanced fabrication and composite strategies further broaden the applicability of fish-derived dECM but also increase design and translational complexity. Reported studies consistently indicate that no single post-processing approach simultaneously optimizes all relevant performance parameters, including mechanical integrity, biological functionality, and process scalability. Instead, favorable outcomes are achieved through application-specific optimization in which material composition, crosslinking density, and fabrication method are tuned to match the biological and mechanical demands of the target tissue.

## 6. Conclusions and Future Perspectives

Fish-derived matrix dECM has emerged as a promising and sustainable biomaterial platform for regenerative medicine and tissue engineering, offering high biocompatibility, biodegradability, and a reduced risk of mammalian pathogen transmission. Through this review, it becomes evident that the biomedical performance of fish-derived dECM is strongly application-dependent and governed by the interplay between intrinsic matrix bioactivity, mechanical requirements, and post-processing strategies.

Soft tissue applications, particularly wound healing and skin regeneration, consistently demonstrate favorable regenerative outcomes due to the biochemical composition of fish skin ECM and relatively low mechanical demands. In contrast, bone and cartilage regeneration highlight critical limitations related to insufficient mechanical stability, accelerated degradation, and loss of key extracellular matrix components following decellularization. These findings indicate that biological compatibility alone is insufficient for functional regeneration in load-bearing tissues and underscore the need for targeted structural reinforcement.

A major challenge limiting broader translation is the lack of standardized decellularization and characterization protocols. Variability in decellularization rate, temperature, detergent selection, and species-specific tissue properties leads to inconsistent scaffold performance and hampers reproducibility. Addressing these gaps will require systematic comparative studies and the adoption of standardized reporting frameworks that link processing parameters to matrix preservation, mechanical behavior, and biological outcomes.

Sterilization and preservation further introduce trade-offs between microbial safety and matrix integrity. While chemical and physical sterilization methods can achieve acceptable sterility assurance levels, many compromise ECM bioactivity or structure. Strategic selection or combination of sterilization approaches remains essential to balance safety with functional performance in clinical applications.

To overcome inherent mechanical limitations, post-processing strategies such as crosslinking, polymer–dECM composites, and biofabrication have become critical enabling tools. While chemical crosslinking improves mechanical stability and degradation resistance, excessive crosslinking can impair porosity and cell infiltration. Polymer–dECM composites and advanced biofabrication approaches provide a more balanced strategy, enhancing structural performance while preserving biological cues, albeit with increased processing and translational complexity.

Collectively, fish-derived dECM should not be viewed as a universal replacement for mammalian ECM, but rather as a context-specific biomaterial whose success depends on rational, application-driven design. Future progress in the field will rely on standardized methodologies, sustainable decellularization strategies, and quantitative structure–property–performance relationships. By integrating these advances, fish waste valorization can bridge sustainability and biomedical innovation, transforming underutilized aquatic byproducts into high-value regenerative biomaterials within a circular bioeconomy framework.

## Figures and Tables

**Table 1:** Chemical Decellularization Protocols for Fish Tissues.

Chemical Treatment	Fish Species	Fish Tissue/Organ	Decellularization Protocol	Results	References
Ionic detergent	*Oreochromis niloticus* (Tilapia)	Scales	Immersed in varying SDS concentrations (0.1%, 0.5%, and 1%) at 4 °C for 72 h, then washed.	Higher SDS concentrations resulted in more protein loss due to its denaturing effect.	[56]
Non-ionic detergent	*Oreochromis niloticus* (Tilapia)	Scales	Immersed in varying TX-100 concentrations (0.1%, 0.5%, and 1%) at 4 °C for 72 h, then washed.	TX-100 was better at preserving the structure and protein content of the dECM than SDS.	[56]
Ionic detergent + Hypotonic solution + Chelating agent	*Sparus aurata* (Gilt-head bream)	Scales	Treated with 0.1% SDS in Tris-HCl buffer and 0.1% EDTA at 4 °C for 3 days.	Fully removed cells, preserved collagen I and porosity (70–78%), as confirmed by SEM and FTIR. Mechanical stability remained high (compression modulus: 13.5–14.3 kPa). No cytotoxic effects were observed, with increasing cell viability and enhanced osteogenic differentiation.	[63]
	*Ctenopharyngodon idella* (Grass carps)	Scales	The sample was incubated in 10 mM Tris-HCl and 0.1% EDTA at 4 °C for 24 h, followed by 0.1% SDS treatment at 4 °C for three days. It was then rinsed five times with distilled water to remove residual chemicals.	SDS treatment led to the complete removal of cells with DNA content less than 50 ng/mg. Hydroxyapatite in the scaffold decreased its content by 55.61%, while there was 55.49% collagen lost. The surface microstructure was destroyed, as shown in SEM. The material exhibited a degradation rate of 49.89 ± 6.56% over eight weeks. The Young’s Modulus decreased significantly to 3.63 ± 2.34 GPa.	[55]
Non-ionic detergent + Hypertonic and hypotonic solutions + Chelating agent	*Ctenopharyngodon idella* (Grass carps)	Skin	Hypertonic treatment (0.5 M NaCl, 25–50 mM Tris, 10 mM EDTA) to break cell membranes, followed by 0.5% TX-100 treatment (24 h) for further cell removal. Finally, the sample was washed with PBS and cold distilled water to eliminate any remaining detergents.	The scaffold had a porous structure (20–100 μm), enhancing cell adhesion and proliferation. SEM confirmed strong epithelial cell attachment, supporting skin regeneration.	[64]
Basic solution + Hypertonic solution	*Oreochromis niloticus* (Tilapia)	Skin	Soaked in 0.1 M NaOH for 8 h, followed by soaking in 1 M NaCl solution for 12 h, then soaked in 0.1 M NaOH again for 6 h.	The scaffold was porous and spongy, with complete cell removal and preserved collagen integrity. Hydroxyproline analysis showed minimal collagen loss, and SEM revealed a two-layered, highly porous structure supporting fibroblast adhesion. FTIR confirmed collagen presence, Calcein AM-PI staining confirmed high cell viability, while mechanical testing revealed a tensile strength of 21.76 MPa.	[65]
Acid solution + Chelating agent	*Carassius auratus* (Goldfish)	Scales	Treated with 5% nitric acid (10 h), and decalcified in 10 wt.% EDTA + 2% nitric acid (3 days, 4 °C, with daily renewal).	Final material was mainly organic (3.5 wt.% inorganic content) with intact fibrillary collagen layers. Cytocompatibility tests showed high cell adhesion and proliferation, with cells aligning along natural ridge channels.	[57]

**Table 2:** Hybrid Physical-Chemical Decellularization Protocols for Fish Tissues.

Physical-Chemical Treatment	Fish Species	Fish Tissue/Organ	Decellularization Protocol	Results	References
Ionic detergent + Agitation	*Thunnus albacares* (Yellowfin Tuna)	Skin	Treated with 0.1% SDS for 2 days at 300 rpm and 4 °C, with solution changes every 24 h, then washed.	H&E staining confirmed significant nuclear loss, while ATR-FTIR showed retention of the collagen triple-helix structure. SEM imaging revealed a smooth inner surface and well-organized collagen fibers, maintaining ECM integrity. DSC analysis indicated thermal stability (71.93 °C).	[57]
	*Chanos chanos* (Milkfish)	Skin	Treated with 0.1% and 1% SDS at 4 °C for 24 h with constant 300 rpm agitation, then washed.	1% SDS achieved the highest DNA removal (3.9 ± 0.65 ng/mg) but caused ECM disruption, with fragmented collagen fibers seen in SEM. Tensile strength dropped (67.22 ± 12.47 MPa), while hydrophilicity increased, aiding cell adhesion.	[53]
	*Oreochromis niloticus* (Tilapia)	Heads	Treated with 0.1% and 1% SDS at 4 °C for 24 h with constant 300 rpm agitation, then washed.	Achieved moderate DNA removal, but higher residual DNA remained compared to sonication-assisted protocols. Collagen structure showed some disruption, and residual SDS levels were high, potentially affecting biocompatibility.	[7]
	*Oreochromis niloticus* (Tilapia)	Viscera	Treated with 1% SDS with agitation for 5 and 10 min. Then washed thrice with distilled water.	0.3% SDS achieved higher DNA removal (94.6% efficiency). However, SDS-treated scaffolds exhibited some ECM disruption, as evidenced by SEM imaging, which showed a denser and more disorganized fiber structure. The mechanical properties declined, exhibiting lower tensile strength and Young’s modulus compared to the raw tissue.	[17]
Non-ionic detergent + Agitation	*Chanos chanos* (Milkfish)	Skin	Treated with 0.1% and 1% TX-100 at 4 °C for 24 h with constant 300 rpm agitation, then washed.	1% SDS preserved the ECM structure, with higher collagen retention and organized fibers. It maintained high tensile strength (118.14 ± 10.27 MPa), had lower residual detergent, and ensured better biocompatibility, though DNA removal was slightly lower than SDS.	[53]
	*Oreochromis niloticus* (Tilapia)	Heads	Treated with 1% TX-100 with agitation for 5 and 10 min. Then washed thrice with distilled water.	1% TX-100 with agitation preserved ECM integrity and porosity better than SDS, though DNA removal was slightly lower. It also had lower residual detergent, improving biocompatibility.	[7]
	*Oreochromis niloticus* (Tilapia)	Viscera	Treated with 0.1% and 0.3% TX-100 with agitation via orbital shaker and magnetic stirrer	TX-100 with agitation preserved ECM integrity better than SDS. At 0.3% concentration, TX-100 maintained a more porous structure with less ECM damage. While DNA removal was slightly lower than SDS, TX-100-treated scaffolds demonstrated better biocompatibility and higher cell adhesion.	[17]
Non-ionic detergent + Hypertonic solution + Basic solution + Agitation	*Oreochromis niloticus* (Tilapia)	Skin	Samples were pretreated with 3% NaCl at 8 °C for 6 h, then placed on filter paper pre-soaked in 0.3% acetic acid. They were subsequently incubated in 0.1% TX-100 at 8 °C for 16 h, followed by 0.1 M NaOH treatment at 8 °C for 6 h with continuous shaking at 150 rpm.	Residual DNA content was 1.4 ± 0.7 ng/mg, well below the 50 ng/mg medical industry limit.	[45]
Freeze–thaw cycle + Acid-base solutions + Ionic detergent	*Oreochromis niloticus* (Tilapia)	Scales	Fish scales were freeze-thawed (−80 °C, 4 cycles), then soaked in 0.1% NaOH and 3% H_2_O_2_. Then, finally, immersed in 2% SDS.	H&E and Masson’s staining confirmed complete decellularization, and SEM showed an intact collagen network. The scaffold retained high tensile strength (5.89 ± 0.74 MPa) and exhibited slow degradation, supporting MSC viability and proliferation, making it biocompatible for tissue regeneration.	[36]
Freeze–thaw cycle + Hypertonic and hypertonic solutions	*Ctenopharyngodon idella* (Grass carps)	Skin	Samples were frozen (−20 °C, 3 cycles, 30 min each) and thawed at room temperature, then treated with a hypertonic solution for 24 h. Finally, incubated in a hypotonic solution for 24 h.	Has high residual DNA content (>100 ng/mg), indicating incomplete decellularization. SEM showed dense collagen fibers, potentially limiting cell infiltration. While mechanical strength remained high (23.15 MPa, 205.8 MPa Young’s modulus), biocompatibility was lower due to remaining cellular debris.	[35]
Freeze–thaw cycle + Hypertonic and hypertonic solutions + Non-ionic detergent	*Ctenopharyngodon idella* (Grass carps)	Skin	Samples were frozen (−20 °C, 3 cycles, 30 min each) and thawed at room temperature, followed by a hypertonic solution for 24 h. Then, treated with a variation in the concentration of TX-100. Finally, incubated in a hypotonic solution for 24 h.	Improved DNA removal (<50 ng/mg in Protocol 4) and collagen preservation, enhancing porosity and cell attachment. Protocol 4 (0.5% TX-100) achieved the best ECM integrity, tensile strength (21.25 MPa), and Young’s modulus (192.15 MPa). High cell adhesion confirmed superior biocompatibility.	[53]
Freeze–thaw cycle + Ionic detergent + Agitation	*Rutilus frisii*	Swim bladder	The fish swim bladder (FSB) was snap freeze-dried by soaking in liquid nitrogen (3 min) followed by rapid thawing in distilled water (5 min). It was then washed in PBS (37 °C) and treated with 0.5% and 1% SDS for 24 h in a shaker incubator.	Histological staining showed the absence of nuclei. SEM imaging revealed a porous structure with collagen fibers intact. The scaffold exhibited high collagen content and a pore size of ~0.5–2 µm, suitable for cell migration. Cytotoxicity tests showed a cell viability of 84–142% after 24 h.	[34]
Sonication + Ionic detergent	*Oreochromis niloticus* (Tilapia)	Heads	Sonication-assisted (40 kHz, 5–10 min) with 1% SDS. Then washed thrice with distilled water.	1% SDS treatment was the most effective in DNA removal (93.7%), with the lowest residual DNA (7.67 ng/mg). However, SEM analysis showed greater ECM disruption, and SDS retention remained high, which may impact cytocompatibility.	[7]
	*Oreochromis niloticus* (Tilapia)	Viscera	Sonication-assisted (40 kHz, 10 h) with 0.1% and 0.3% SDS at room temperature. Then washed 3 times.	Sonication enhanced DNA removal, with 0.3% SDS achieving 96.5% efficiency. It accelerated decellularization, but SEM revealed greater ECM disruption, showing fragmented collagen fibers and rougher surfaces.	[17]
Sonication + Non-ionic detergent	*Oreochromis niloticus* (Tilapia)	Heads	Sonication-assisted (40 kHz, 5–10 min) with 1% TX-100. Then washed thrice with distilled water.	1% TX-100 also achieved high DNA removal, but better collagen preservation and porosity retention than SDS-treated samples. Additionally, residual TX-100 was significantly reduced (99% lower for 5 min treatment), enhancing biocompatibility.	[7]
	*Oreochromis niloticus* (Tilapia)	Viscera	Sonication-assisted (40 kHz, 10 h) with 0.1% and 0.3% TX-100 at room temperature. Then washed 3 times.	Sonication with 0.3% TX-100-preserved ECM integrity while ensuring high DNA removal. Collagen remained more intact than in SDS-treated samples, with better cell viability and attachment, offering a balance between decellularization and ECM preservation.	[17]

**Table 3:** Hybrid Chemical–Biological Decellularization Protocols for Fish Tissues.

Physical-Chemical Treatment	Fish Species	Fish Tissue/Organ	Decellularization Protocol	Results	References
Ionic detergent + Enzyme + Chelating agent	Sturgeon fish	Cartilage	Cartilage samples were treated with 1% SDS in PBS (4 °C, 24 h) with solution changes every 8 h, followed by 0.1% EDTA in PBS (24 h). They were then digested in 1 U/mL DNase I (24 h) and washed in PBS.	The H&E staining showed no visible nuclear remnants, indicating effective decellularization.	[58]
Non-Ionic detergent + Enzyme + Hypotonic solution + Chelating agent	*Ctenopharyngodon idella* (Grass carps)	Scales	The samples were incubated in 10 mM Tris-HCl and 0.1% EDTA at 4 °C for 24 h, followed by 0.1% TX-100 treatment at 4 °C. They were then digested with a nuclease solution containing 500 U/mL DNase I and 1 mg/mL RNase A at 37 °C for 24 h.	TX-100 treatment for 3 days could not completely remove the cellular components of the scaffolds, while extending the treatment time to 4 and 5 days completely removed them. Treatment for 3 days and 4 days has better effects in retaining ECM components while preserving the intact surface microstructure. The degradation rate of 3- and 4-day treatments was also lower and had no significant difference between them. Their Young’s modulus decreased slightly to 6.26 ± 1.49 and 5.80 ± 1.17 GPa. In general, the 4-day treatment proved to be the best protocol.	[55]
Ionic detergent + Non-Ionic detergent + Enzyme + Hypotonic solution	*Oreochromis niloticus* (Tilapia)	Skin	Samples were incubated in PBS with 0.02% sodium azide and 0.5% TX-100 (RT, 2 h), washed with HBSS (RT, 10 min), treated with 0.5% SDS (RT, 1 h), and digested in trypsin (0.05 g/mL) with 1 M Tris-HCl.	H&E staining confirmed that the decellularization process effectively removed cells, increased scaffold porosity, and preserved the ECM’s original structure. Mechanical testing showed that the AFS scaffold exhibited high tensile strength and flexibility. Degradation studies revealed that approximately 70% of the matrix degraded within 28 days, exhibiting behavior similar to collagen-based scaffolds.	[70]

**Table 4:** Hybrid Physical-Chemical-Biological Decellularization Approaches for Fish Tissues.

Physical-Chemical Treatment	Fish Species	Fish Tissue/Organ	Decellularization Protocol	Results	References
Freeze–thaw cycle + Non-ionic detergent + Hypertonic and hypotonic solutions + Chelating agent + Enzyme	*Ctenopharyngodon idella* (Grass carps)	Skin	Samples were frozen (−20 °C, 3 cycles, 30 min each) and thawed at room temperature, then treated with a hypertonic solution for 24 h. Followed by a variation in concentration of Trypsin-EDTA for 90 min. Then, TX-100 treatment was performed at varying concentrations. Finally, incubated in a hypotonic solution for 24 h.	Trypsin-EDTA (0.25%) enhanced decellularization (DNA < 20 ng/mg in Protocol 6) but caused greater ECM disruption, reducing collagen content, mechanical strength, and cell adhesion. Weaker scaffolds degraded faster in PBS, compromising stability despite effective cell removal.	[35]
Freeze–thaw cycle + Non-ionic detergent + Enzymes in Hypotonic solution + Agitation	*Mylopharyngodon piceus* (Black carps)	Skin	Samples were frozen at −40 °C, thawed in tap water, and stirred in 1% TX-100 (12 h) and 0.5 μg/mL trypsin (18 h, pH 8.0 Tris–HCl buffer) for effective decellularization while preserving the extracellular matrix.	H&E and SEM confirmed a cell-free, porous AFS with preserved 3D structure and well-arranged collagen fibers. Cytotoxicity tests showed no toxicity to L929 cells. AFS exhibited super hydrophilicity, a swelling ratio that stabilized by day 5, and slow degradation over 13 weeks with high wet-condition resistance (0.07 ± 0.04%).	[33]
Freeze–thaw cycle + Ionic detergents + Enzyme + Agitation	*Hypopththalmichthys molitrix* (Chubs)	Swim bladder	The SBs underwent three freeze–thaw cycles (−80 °C, 1 h; 36 °C thaw), followed by detergent treatment (0.5% sodium deoxycholate, 0.05% sodium orthovanadate in PBS, RT, 12 h, 110 r/min) and DNase-I treatment (20 U/mL, 37 °C, 2 h, shaking).	The freeze–thaw and DNase-I treatment (Group E) was the most effective for decellularization, yielding low residual DNA. The decellularized ASBs had a loosened fiber layer and 3D porous structure, promoting cell adhesion and migration. Additionally, the hemolysis rate (2.8 ± 0.15%) was well below the 5% threshold, confirming good biocompatibility for medical applications.	[39]
Freeze–thaw cycles + Ionic detergent + Enzyme	*Astroconger Myriaster*	Skin	The samples underwent three freeze–thaw cycles (−80 °C), followed by 2.0% deoxycholic acid treatment (12 h) for decellularization. They were then rinsed and treated with 0.5% SDS (1 h) and subjected to enzymatic digestion with 0.2 μg/L trypsin (4 h).	Cellular components were successfully removed as confirmed by H&E, Masson, and PAS staining, leaving a collagen-rich matrix with no PAS-positive substances. DNA content was reduced to 18.64 ± 2.51 ng/mg, significantly lower than natural fish skin (234.17 ± 13.24 ng/mg) and commercial oral membranes (32.41 ± 4.87 ng/mg), indicating effective decellularization.	[59]
Non-ionic detergent + Hypotonic solution + Enzyme + Agitation	Crisp flesh grass carp	Scales	Fish scales were stirred in Tris-buffer with PMSF (4 °C, 36 h), treated with 1% TX-100 (4 °C, 36 h), then digested with DNase/RNase (37 °C, 2 h) and extracted in TX-100 (4 °C, 24 h).	Cells were effectively removed (DNA: 23.5 ± 1.8 ng/mg) while preserving ECM integrity. H&E and DAPI staining confirmed complete cell removal, and SEM showed a porous collagen network. The scaffold retained high tensile strength (92.7 ± 6.3 MPa) and Young’s modulus (2.5 ± 0.4 GPa). Degradation studies showed 54.2% weight retention (28 days, PBS), and cell viability (>90%) confirmed high biocompatibility.	[52]
Ionic detergent + Enzymes + Agitation	*Oreochromis niloticus* (Tilapia)	Skin	The skin samples were shaken in 2.5 U/mL dispase (3 h), followed by 1% SDS treatment (6 h) and gentle scraping. They were then shaken in 25 U/mL nuclease (3 h), treated with 1% SDS (1 h), and finally freeze-dried.	Decellularization removed pigments, preserved 69.3% collagen, and loosened fibers while maintaining structure. SEM showed increased porosity, strong cell attachment, and minimal surface changes. Tensile strength decreased, but thermal stability remained high (>60 °C). Degradation: 81.0% retained in PBS (8 weeks), 13.7% in collagenase (72 h).	[66]

**Table 5:** Strategic Selection and Optimization of Fish-Derived dECM Protocols.

Fish-Tissue Type	Best-Performing Protocol Families	Key Parameters	Typical Pitfalls	Recommended Characterization Endpoints
Skin	Basic-Hypertonic Sequence or Mild Chemical	0.1 M NaOH (8 h)/1 M NaCl (12 h); or 0.5% TX-100	High lipid content in skin can hinder the penetration of aqueous reagents; SDS risks fiber orientation disruption.	H&E for nuclear loss, ATR-FTIR for triple-helix integrity
Scales	Hybrid: Non-ionic detergent + Chelating agent + Nuclease	0.1% TX-100 + 10 mM EDTA; 4-day incubation at 4 °C	SDS treatment (0.1%) causes ~55% collagen loss and destroys surface microstructure.	DNA quantification (<50 ng/mg), SEM for porosity (70–78%)
Viscera/Soft organs	Physical-assisted Chemical: Sonication + Detergent	40 kHz sonication (10 min); 0.3% SDS	Higher protein loss is observed in sonicated samples; requires strict control of burst times to prevent thermal denaturation.	DNA removal efficiency (>95%), DSC for thermal stability (>60 °C)
Bones	Acid-Chelating: Acidic demineralization + EDTA	5% Nitric acid (10 h); 10% EDTA (3 days, 4 °C)	Acidic hydrolysis can lead to mechanical weakening if exposure is too long.	Residual inorganic content wt.%, SEM for cell-alignment channels
Swim Bladder	Mild Chemical + Crosslinking	Low-concentration TX-100; Post-process with GA or EDC/NHS	High mechanical fragility; susceptible to tearing during agitation or rinsing.	Degradation rate and porosity analysis

**Table 6:** Biomedical Performance of Fish-Derived dECM Across Regenerative Medicine Applications.

Biomedical Application	Biological Property Assessed	Observations Reported	Fish Source and Tissue	Observed Biological Advantages	References
Wound healing/Skin regeneration	Cell adhesion and proliferation	Enhanced fibroblast and keratinocyte adhesion and proliferation reported when collagen fibrillar structure and GAG content are preserved	Tilapia, cod, salmon skin	Strongly dependent on detergent type, exposure time, and tissue thickness	[13,16,73,76,77]
	Angiogenic response	Increased neovascularization observed in select in vivo wound models; magnitude varied across studies	Fish skin ECM	Sensitive to GAG retention and lipid preservation during decellularization	[13,16,33,75,78,79]
	Inflammatory response	Reduced inflammatory cell infiltration reported under minimally processed conditions; variable outcomes reported following aggressive chemical treatments	Fish skin grafts	Influenced by residual detergent content and matrix disruption	[13,16,75,80,81]
Bone regeneration	Osteogenic differentiation	Upregulation of osteogenic markers and mineral deposition observed in vitro	Fish scale- and bone-derived dECM	Sensitive to mineral preservation and collagen integrity	[75,82,83,84]
	Structural integrity under load	Limited mechanical stability reported without secondary reinforcement	Fish-derived demineralized bone matrix (DBM)	Strongly affected by decalcification and processing severity	[13,52,85,86,87,88,89,90,91,92]
Cartilage repair	Chondrogenic response	Chondrocyte attachment and differentiation supported; suppression of hypertrophic markers reported in select studies	Sturgeon cartilage dECM	Sensitive to GAG depletion during decellularization	[40,75,93,94,95]
	Compressive performance	Reduced compressive properties reported following loss of sulfated GAGs	Fish cartilage dECM	Strong dependence on decellularization chemistry	[58,93]

**Table 7:** Effects of Post-Processing Strategies on the Performance of Fish-Derived dECM.

Strategy	Specific Method/Material	Targeted Limitation	Reported Qualitative Effect	Trade-Offs/Limitations	References
Crosslinking	EDC/NHS	Mechanical weakness	↑ tensile strength; ↓ degradation rate	Reduced porosity at high density	[89,117,118,119,120,121,122]
	DHT	Structural instability	↑ thermal stability	Limited tunability	[89,120,123,124]
Polymer composite	dECM–Alg/MA	Poor printability	↑ shape fidelity; ↑ porosity	Polymer dilution of ECM cues	[125,126,127,128,129]
	dECM–GelMA	Weak mechanics	↑ modulus; ↑ cell viability	UV exposure concerns	[126,130,131,132]
Nanocomposite	Collagen nanofibers	Low strength	↑ mechanical reinforcement	Scale-up challenges	[133,134]
	3D Printing (dECM bio-inks)	Low viscosity; Poor print fidelity	↑ shape fidelity; ↑ precise microarchitecture control; ↑ preserved bioactivity	Complexity of ink formulation and dependency on crosslinking/additives	[32,105,125,132,135,136]

## Data Availability

No new data were created or analyzed in this study.
